# Alien Chromatin from Hordeeae Grasses Enhances the Compatibility of *Epichloë* Endophyte Symbiosis with the Hexaploid Wheat *Triticum aestivum*

**DOI:** 10.3390/jof10060384

**Published:** 2024-05-27

**Authors:** Wayne R. Simpson, Hisashi Tsujimoto, David E. Hume, Richard D. Johnson

**Affiliations:** 1 AgResearch, Grasslands Research Centre, Private Bag 11008, Palmerston North 4442, New Zealand; david.hume@agresearch.co.nz; 2 Arid Land Research Center, Tottori University, 1390 Hamasaka, Tottori 680-0001, Japan; tsujim@tottori-u.ac.jp

**Keywords:** *Epichloë*, symbiosis, wheat, *Triticum aestivum*, alien chromosome, Triticeae, Hordeeae

## Abstract

The inoculation of *Epichloë* endophytes into modern cereals, resulting in systemic infection, depends on the genetics of both the host and the endophyte strain deployed. Until very recently, the only modern cereal to have been infected with *Epichloë*, in which normal phenotype seed-transmitted associations were achieved, is rye (*Secale cereale*). Whilst minor in-roads have been achieved in infecting hexaploid wheat (*Triticum aestivum*), the phenotypes of these associations have all been extremely poor, including host death and stunting. To identify host genetic factors that may impact the compatibility of *Epichloë* infection in wheat, wheat–alien chromosome addition/substitution lines were inoculated with *Epichloë*, and the phenotypes of infected plants were assessed. Symbioses were identified whereby infected wheat plants were phenotypically like uninfected controls. These plants completed their full lifecycle, including the vertical transmission of *Epichloë* into the next generation of grain, and represent the first ever compatible wheat–*Epichloë* associations to be created.

## 1. Introduction

*Epichloë* endophytes, particularly asexual forms, have important roles in pastoral agricultural systems in the Americas, Australia and New Zealand [1]. Selected strains add value to grass-based forage systems by providing both biotic and abiotic stress resistance. The symbioses of anamorph-typified *Epichloë* form are mutualistic in that both the fungus and the host grass benefit from the association. The fungus benefits from a biological niche with few if any competing organisms, a source of nutrients in the host’s apoplastic fluid and a mechanism for vicarious dispersal via the host seed. The host benefits from the range of secondary metabolites the fungus produces in the form of alkaloids, many of which have individual and/or multiple activities against different classes of organism [1].

Modern Hordeeae (Triticeae) cereal grasses such as wheat (*Triticum aestivum*), barley (*Hordeum vulgare*) and rye *(Secale cereale*) do not host *Epichloë* endophytes, although grasses of some genera within the tribe, such as *Elymus*, *Hordeum* and *Leymus,* do [2,3,4,5,6,7,8,9].

Both organism classes, *Epichloë* endophytes and cereal grasses, are of great importance in their own contexts, and the prospect of forming functioning symbioses offers the potential to improve cereal production systems. Previous research [10] demonstrated varying outcomes of symbiosis. Whereas symbioses established with rye (*Secale cereale*) showed phenotypes ranging from normal to compromised, those involving modern hexaploid wheat (*Triticum aestivum*) were always poor, exhibiting a dwarfed phenotype. This likely reflects that outcrossing species such as rye present a range of genotypes in an inoculated population, whereas inbred species such as wheat are a genetically narrow host population target.

Wild relatives of wheat are recognized as having potential as sources of genes for improving wheat performance; for example, the introduction of pathogen resistance using rye chromatin [11]. The best-known example of interspecific chromatin being transferred into a crop is the translocation of rye chromosome arm 1RS to wheat chromosome arm 1BL [12]. It is possible to add or substitute entire chromosomes, chromosome areas or chromatin segments. The rearrangement of wheat chromosomes in this way has constituted an important aspect of wheat improvement for over 50 years [13]. The transfer is effected by producing an amphidiploid, a hybrid between the two species with at least one complete diploid set of chromosomes from each species, or a partial amphidiploid, and then producing individual chromosome addition lines. Following this, the centric breakage fusion tendency can be exploited to transfer a whole alien chromosome arm. Strategies can also be deployed for transferring alien segments that are smaller than complete chromosome arms [14]. Where genetic diversity is limited, such as in highly selected and inbred wheat lines, the introduction of alien genetics from related species offers a means to broaden the genetic base of the population and increase the possibilities for the selection of desirable traits [15,16,17].

To improve the outcome of synthetic *Epichloë* symbioses with wheat, we utilized alien chromosome additions and substitutions from both rye and wild grass species from within the Hordeeae tribe (Supplementary Table S1).

## 2. Materials and Methods

### 2.1. Endophyte Strain

*Epichloë bromicola* AR3060 (AgResearch strain 3060) was isolated from an *Elymus dahuricus* subspecies *excelsus* plant and used in inoculation experiments in Japan following isolation from the parent plant as previously described [18]. *Epichloë bromicola* AR3002 was isolated from an *Elymus dahuricus* plant and used in inoculation experiments in New Zealand. Fungal cultures were grown on potato dextrose agar as described by Fleetwood et al., 2007 [19].

### 2.2. Inoculation of Wheat Alien Chromosome Substitution and Addition Lines

Experiments involving the inoculation of wheat lines sourced from the Tottori Alien Chromosome Bank Of Wheat (TACBOW) at the National BioResource Project (NBRP) Komugiwere performed as follows.

Up to five seeds from each of 155 wheat lines with various alien chromosome introductions from species of *Aegilops*, *Agropyron*, *Elymus*, *Hordeum*, *Leymus*, *Psathyrostachys*, *Haynaldia* and *Secale*, along with the wheat cultivars ‘Monad’ and ‘Chinese Spring’ as controls (Supplementary Table S1), were inoculated, as described by Latch and Christensen (1985) [20], using strain AR3060, which had previously been demonstrated to infect the wheat cultivar “Monad”, albeit with a compromised host phenotype [10]. Plants were grown for ca. 6 weeks in commercial potting mix before identifying infected individuals through tissue-print immunoblotting as previously described [18]. Plants that were demonstrated to be infected with *Epichloë* by immunoblot were grown for a further 3 months under glasshouse conditions to complete their full life cycle. Endophyte-free plants of the same lines were also grown under equivalent conditions to serve as direct comparisons to the infected plants.

In subsequent experiments performed in Palmerston North, New Zealand, a subset of lines (Supplementary Table S2) that supported compatible AR3060 infection were inoculated with *E. bromicola* strain AR3002, as, unlike AR3060, this strain does not produce the mammalian toxin ergovaline.

### 2.3. Phenotyping

Plants were phenotyped at the completion of their life cycle and those that were originally infected, based upon earlier immunoblotting results, were confirmed as remaining *Epichloë* infected by microscopy and/or fungal isolation, as described below. Infected individuals were phenotypically compared to plants of the same line that did not become infected.

### 2.4. Epidermal Leaf Peel

Tillers were selected from mature plants for endophyte detection. Clean, live sheath tissue was placed under a dissecting microscope at 16× magnification with the adaxial epidermis facing up. A shallow transverse cut was made with a scalpel and the epidermis was gently lifted, separated, and pulled off the sheath. Tissue was mounted in a drop of aniline blue dye (glycerol 50%; lactic acid 25%; water 24.95%; aniline blue 0.05%), heated over a naked flame and examined at 100× and 400× using a compound microscope.

### 2.5. Seed Squash

Grain was covered with a 5% sodium hydroxide solution and left to imbibe overnight. The following day, the solution was decanted and the samples thoroughly rinsed with tap water. Samples were covered with Garner’s solution (0.325 g aniline blue, 100 mL water and 50 mL 85% lactic acid) and heated to boiling on a hot plate. After cooling, the softened grain was mounted on a microscope slide, a cover slip was placed over the grain and gentle, even pressure was applied, squashing the preparation. The preparations were then examined under a compound light microscope at 100× and 400× magnification.

### 2.6. Fungal Isolation

Fungus was isolated from plants following surface sterilization of plant tissue, as described by Christensen, et al. (2002) [21]. Blade tissue was surface sterilized by quick rinse with 96% ethanol and a 1 min soak in a sodium hypochlorite solution (10% Janola: 42 g/L NaOCl domestic bleach), followed by rinsing twice in sterile water. Tissue was plated on to potato dextrose agar containing 5 µg/mL tetracycline. Plates were incubated at 22–25 °C for 3–5 days.

## 3. Results and Discussion

### Epichloë Infection of Wheat Alien Chromosome Addition/Substitution Lines

**Figure 1:**
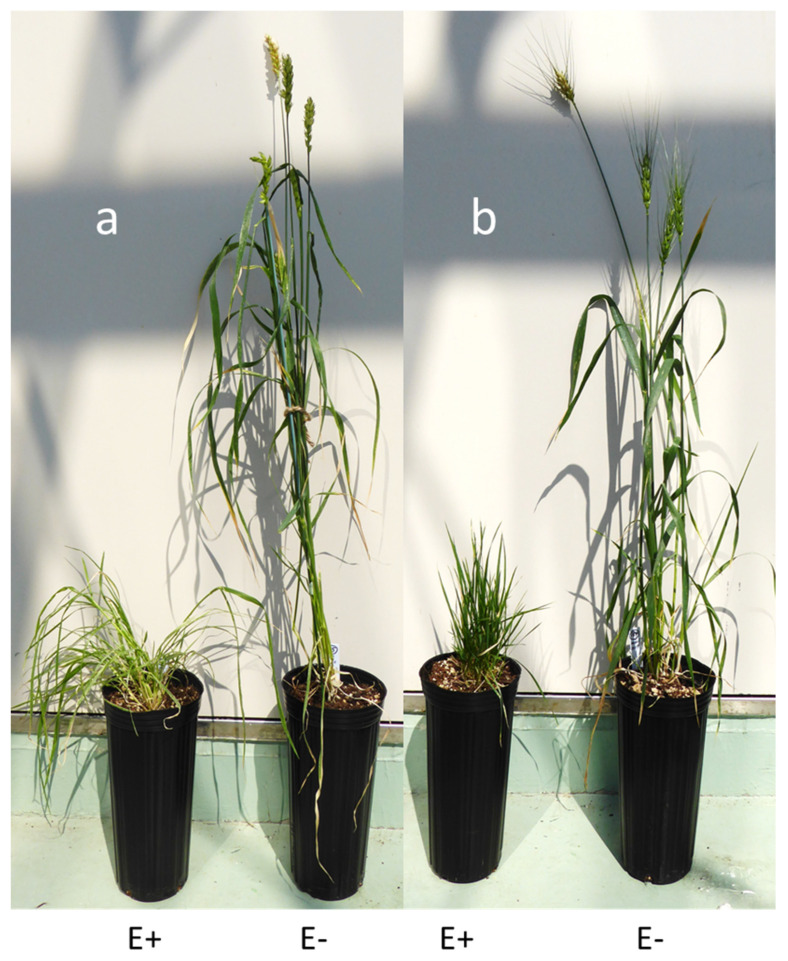
Wheat cultivars (**a**) Chinese Spring and (**b**) Monad, infected (E+) or uninfected (E−) with *Epichloë bromicola* strain AR3060.

Of the 157 lines inoculated, immunoblot results showed that 97 lines had one or more *Epichloë*-infected plants (Table 1), with the remaining lines being uninfected. All lines were subsequently phenotyped at maturity. Uninfected lines were universally tall and floral with fully developed grain and, significantly, wheat lines with no alien chromosome introductions (Chinese Spring and Monad) were dwarfed (Figure 1) in a similar manner to that previously reported [10]. Infected plants of chromosome addition/substitution lines included tall floral individuals that were similar to uninfected plants of the same line and abnormal phenotype plants that were short in stature and often failed to produce inflorescences. Additionally, tall floral plants delayed in their maturation that were exemplified by a ‘stay green’ phenotype and a protracted elaboration and maturation of the floral spike were identified. Additional evidence for endophyte infection was obtained for a sub-set of plants from each of the three infection phenotypes using a combination of fungal isolation and microscopy of leaf and seed material. Although not all the plants that had a delayed maturation were confirmed to be infected in this way, this phenotype was indicative of endophyte infection and plants were classified as such for further analysis.

**Figure 2:**
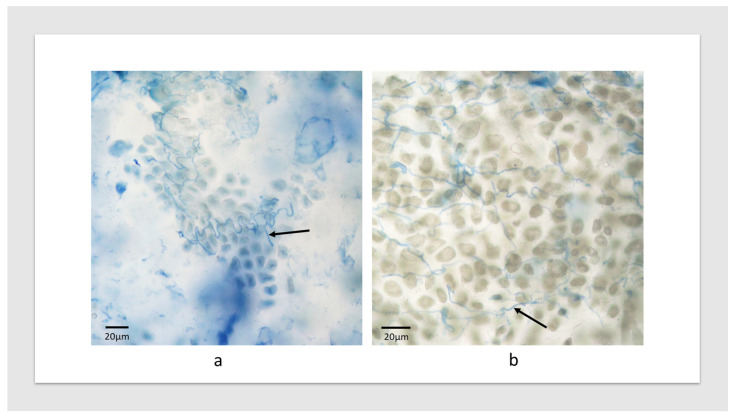
Seed of (**a**) TACBOW11 and (**b**) TACBOW67 infected with *Epichloë bromicola* strain AR3060. Arrows indicate stained hyphae.

An analysis of the alien genetics associated with TACBOW lines displaying tall floral infection phenotypes (Table 1) was performed in terms of the donor species and which of the seven homoeologous groups (based upon wheat) the chromosome belonged to, aiming to reveal patterns in host genetics associated with the phenotypic outcomes. We initially looked specifically at the full wheat–rye chromosome addition lines comprising seven “Chinese Spring”-based lines with 1R-7R additions from *S. cereale*. Tall floral infected plants were obtained for wheat–rye 1R, 3R and 7R addition lines, with abnormal phenotypes or no infection occurring with the remaining four addition lines (2R, 4R, 5R, 6R). This result suggests that there may be genetic factors associated with alien chromosomes from homoeologous groups 1, 3 and 7 and, indeed, chromosome additions or substitutions from other grasses, distinct from rye, with these homoeologous groups were also enriched for tall floral infection phenotypes (Table 1). For example, the inoculation of the line TACBOW11, a line with alien genetics representing homoeologous group 3, produced tall floral infected plants. The underlying genetics of this line is *T. aestivum* cv. Chinese Spring *Leymus racemosus* H substitution (20″ + 1″ [H]). Line TACBOW5 was another example of tall floral infected plants involving the *L. racemosus* H chromosome, but as an addition rather than a substitution (21″ + 1″ [H]). Line TACBOW7 also gave rise to infected large phenotype plants but with a delayed maturation. Despite this, grain was fully filled and was confirmed to contain endophyte by microscopy (Figure 2). The underlying genetics of this line is *T. aestivum* cv. Chinese Spring *L. racemosus* J addition (20″ + 1″ [J]). *Leymus* J and *Leymus* H chromosomes have been shown, using RFLP markers, to share some syntenic regions [17,22], which also have homology with wheat chromosomes belonging to homoeologous groups 1, 3 and 7. Interestingly, chromosomes 1, 3 and 7 have been described as sharing a “close genetic relationship” [23] (pp. 29–45) and [24] (p. 73).

Several lines were identified in which, like the *Leymus* J addition line described above, maturation was somewhat delayed. Some of these lines were confirmed as endophytes infected by isolations, leaf sheath microscopy or seed squash (Table 1). The remainder are assumed to be infected, as uninfected lines were universally tall and floral, with fully developed grain. These delayed phenotype lines have certain genetics in common with each other and to those plants showing normal, mature seed-transmitting phenotypes. As described above, the *Leymus* H and J chromosomes share some synteny, and with respect to wheat, share homology with chromosomes belonging to homoeologous groups 1, 3 and 7. In addition to the *Leymus H* (substitution) and *Leymus* J (addition) lines, a *Leymus* N line was also confirmed to be infected (by isolation) and the *Leymus* N chromosome also shares homology with homoeologous groups 3 and 7. Further to this, we confirmed that many of the delayed maturation infection phenotypes also contain alien additions from homoeologous groups 1, 3 or 7 (Table 1). These include, in addition to the 1R, 3R and 7R additions from *Secale* described above, the 1U, 3U and 7U additions from *Aegilops* and 1H and 7H additions from *Elymus*. Whilst these lines are not exclusive and the evolution of the Hordeeae is complex, there seems to be some evidence that genes from wild species located on chromosomes belonging to homoeologous groups 1, 3 and 7 may be beneficial in terms of their compatibility with *Epichloë*.

The ability to infect rye with *Epichloë* and obtain normal phenotype plants [10], together with TACBOW lines involving rye 1R, 3R and 7R chromosome additions (this study), suggests that wheat–rye hybrids might also contain the genetics required to form floral associations with *Epichloë.* Whilst some limited attempts have been made to infect commercial Triticale (*T. aestivum* × *S. cereale*) lines (Simpson, unpublished), in the present study, two *T. turgidum* × *S. cereale* amphidiploid lines (TACBOW66 and TACBOW67) containing all seven rye chromosomes became infected and produced inflorescences.

**Figure 3:**
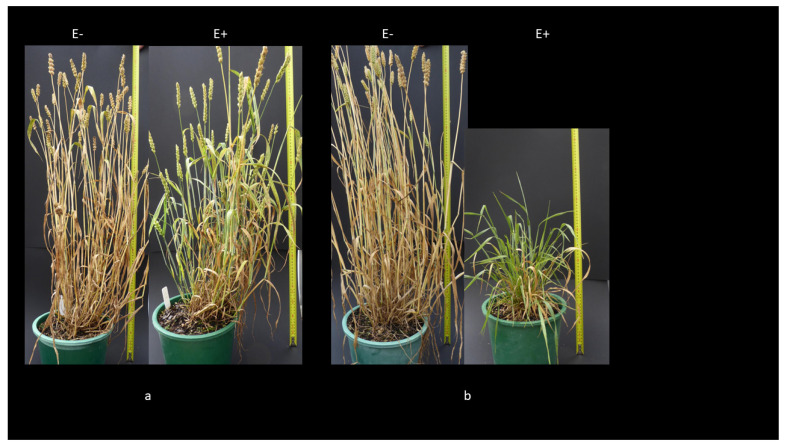
(**a**) TACBOW232- and (**b**) TACBOW288-infected (E+) and uninfected (E−) with *Epichloë bromicola* strain AR3002.

In subsequent inoculations, performed in New Zealand, with the non-toxic strain AR3002 into a sub-set of TACBOW lines, we also saw a range of infection phenotypes. Examples of these are shown in (Figure 3) and plants were confirmed by seed squash to be transmitting *Epichloë* into the grain.

Whether the host genetics required for *Epichloë* compatibility will apply to all *E. bromicola* strains beyond those described here remains to be tested, as functional differences based upon the *Epichloë* genotype have been observed (unpublished data). Regardless, the genetics underlying *Epichloë* compatibility needs to be introgressed into modern cultivars since Chinese Spring is not agronomically useful.

## 4. Conclusions

The inoculation of commercial hexaploid wheat with *Epichloë* has, to date, been unsuccessful, with the resulting infected plants having poor phenotypes. This result was confirmed here with inoculations of the wheat cultivars Chinese Spring and Monad (no chromosome additions or substitutions). However, in this study, the inoculation of Chinese Spring-based wheat containing certain alien chromosome additions or substitutions sourced from rye and wild grass species (many of which also host *Epichloë*) were successful. This suggests that lines containing chromosome additions/substitutions, where infection produced tall floral plants, may have a genetic profile underlying this. Our analysis of these lines indicates that alien chromosome introgressions from homoeologous groups 1, 3 and 7 may be important for the improved compatibility between wheat and *Epichloë*. However, whilst infected normal phenotype lines were enriched for 1, 3 and 7 chromosome additions/substitutions, some infected plants displaying normal phenotypes consisted of lines carrying chromosome additions that did not belong to homoeologous groups 1, 3 or 7. Further work will be required to prove the role of 1, 3, 7 homoeologous group genetics and to determine if there are other commonalties present on some of the non-1, -3 or -7 chromosome groups where infection phenotypes were not compromised.

## 5. Patents

A patent (International application number PCT/IB2019/051395) resulted from the work reported in this manuscript.

## Figures and Tables

**Table 1:** Wheat lines, alien introgressions and *Epichloë*-infection phenotypes.

Line	Wheat Genetic Background	Alien Introgression	Infection Phenotype Class
Chinese Spring	*Triticum aestivum* cv. Chinese Spring	None	dwarfed
Monad	*Triticum aestivum* cv. Monad	None	dwarfed
TACBOW0001	*Triticum aestivum* cv. Chinese Spring	*Leymus racemosus* A addition	dwarfed
TACBOW0003	*Triticum aestivum* cv. Chinese Spring	*Leymus racemosus* E monosomic addition	tall floral
TACBOW0005	*Triticum aestivum* cv. Chinese Spring	*Leymus racemosus* H addition	tall floral
TACBOW0007	*Triticum aestivum* cv. Chinese Spring	*Leymus racemosus* J addition	tall floral ^1^
TACBOW0008	*Triticum aestivum* cv. Chinese Spring	*Leymus racemosus* K addition	tall floral ^1^
TACBOW0009	*Triticum aestivum* cv. Chinese Spring	*Leymus racemosus* l addition	tall floral ^1^
TACBOW0010	*Triticum aestivum* cv. Chinese Spring	*Leymus racemosus* N addition	tall floral
TACBOW0011	*Triticum aestivum* cv. Chinese Spring	*Leymus racemosus* H substitution	tall floral
TACBOW0012	*Triticum aestivum* cv. Chinese Spring	*Leymus racemosus* 2Lr#1 addition	dwarfed
TACBOW0015	*Triticum aestivum* cv. Chinese Spring	*Leymus racemosus* 7Lr#1 addition	dwarfed
TACBOW0016	*Triticum aestivum* cv. Chinese Spring	*Leymus racemosus* ?Lr#1 addition	dwarfed
TACBOW0017	*Triticum aestivum* cv. Chinese Spring	*Leymus racemosus* 2Lr#1 substitution	dwarfed
TACBOW0018	*Triticum aestivum* cv. Chinese Spring	*Secale cereale* cv. Imperial 1R addition	tall floral
TACBOW0020	*Triticum aestivum* cv. Chinese Spring	*Secale cereale* cv. Imperial 3R addition	tall floral ^1^
TACBOW0024	*Triticum aestivum* cv. Chinese Spring	*Secale cereale* cv. Imperial 7R addition	tall floral ^1^
TACBOW0025	*Triticum aestivum* cv. Chinese Spring	*Secale cereale* cv. IR130 1R addition	tall floral ^1^
TACBOW0026	*Triticum aestivum* cv. Chinese Spring	*Secale cereale* cv. IR130 1R substitution	dwarfed
TACBOW0028	*Triticum aestivum* cv. Chinese Spring	*Secale cereale* cv. IR130 3R addition	tall floral
TACBOW0032	*Triticum aestivum* cv. Vilmorin 27	*Thinopyrum intermedium* 7Ai addition	dwarfed
TACBOW0034	*Triticum aestivum* cv. Vilmorin 28	*Thinopyrum intermedium* 1Ai addition	dwarfed
TACBOW0036	*Triticum aestivum* cv. Vilmorin 29	*Thinopyrum intermedium* 5Ai addition	dwarfed
TACBOW0038	*Triticum aestivum* cv. Chinese Spring	*Thinopyrum elongatum* 1E addition	tall floral ^1^
TACBOW0040	*Triticum aestivum* cv. Chinese Spring	*Thinopyrum elongatum* 3E addition	dwarfed
TACBOW0041	*Triticum aestivum* cv. Chinese Spring	*Thinopyrum elongatum* 4E addition	dwarfed
TACBOW0043	*Triticum aestivum* cv. Chinese Spring	*Thinopyrum elongatum* 5E addition	dwarfed
TACBOW0045	*Triticum aestivum* cv. Chinese Spring	*Aegilops umbellulata* 1U addition	tall floral
TACBOW0046	*Triticum aestivum* cv. Chinese Spring	*Aegilops umbellulata* 2U addition	dwarfed
TACBOW0047	*Triticum aestivum* cv. Chinese Spring	*Aegilops umbellulata* 4U addition	dwarfed
TACBOW0048	*Triticum aestivum* cv. Chinese Spring	*Aegilops umbellulata* 5U addition	dwarfed
TACBOW0049	*Triticum aestivum* cv. Chinese Spring	*Aegilops umbellulata* 6U addition	dwarfed
TACBOW0052	*Triticum aestivum* cv. Chinese Spring	*Hordeum chilense* 2H^ch^S addition	tall floral ^1^
TACBOW0053	*Triticum aestivum* cv. Chinese Spring	*Hordeum chilense* 4H^ch^ addition	tall floral ^1^
TACBOW0054	*Triticum aestivum* cv. Chinese Spring	*Hordeum chilense* 5H^ch^ addition	tall floral
TACBOW0055	*Triticum aestivum* cv. Chinese Spring	*Hordeum chilense* 6H^ch^ addition	dwarfed
TACBOW0056	*Triticum aestivum* cv. Chinese Spring	*Hordeum chilense* 7H^ch^ addition	tall floral ^1^
TACBOW0057	*Triticum aestivum*	*Dasypirum villosum* 1V addition	dwarfed
TACBOW0059	*Triticum aestivum*	*Dasypirum villosum* 3V addition	tall floral
TACBOW0064	*Triticum aestivum* cv. Chinese Spring	*T. aestivum-Secale cereale* cv. Imperial amphidiploid	tall floral ^1^
TACBOW0067	*Triticum turgidum*	*T. turgidum-Secale cereale* amphidiploid	tall floral ^1^
TACBOW0124	*Triticum aestivum* cv. Chinese Spring	*Leymus mollis* G addition	tall floral ^1^
TACBOW0125	*Triticum aestivum* cv. Chinese Spring	*Leymus mollis* H addition	dwarfed
TACBOW0126	*Triticum aestivum* cv. Chinese Spring	*Psathyrostachys huashanica* A addition	dwarfed
TACBOW0128	*Triticum aestivum* cv. Chinese Spring	*Psathyrostachys huashanica* C addition	tall floral
TACBOW0138	*Triticum aestivum* cv. Chinese Spring	*Thynopyrum intermedium* G addition	dwarfed
TACBOW0189	*Triticum aestivum* cv. Alcedo	*Aegilops markgrafii* B addition	dwarfed
TACBOW0190	*Triticum aestivum* cv. Alcedo	*Aegilops markgrafii* C addition	dwarfed
TACBOW0193	*Triticum aestivum* cv. Alcedo	*Aegilops markgrafii* E addition	dwarfed
TACBOW0195	*Triticum aestivum* cv. Chinese Spring	*Aegilops longissima* 1S^l^ addition	dwarfed
TACBOW0196	*Triticum aestivum* cv. Chinese Spring	*Aegilops longissima* 2S^l^ addition	tall floral ^1^
TACBOW0197	*Triticum aestivum* cv. Chinese Spring	*Aegilops longissima* 3S^l^ addition	tall floral ^1^
TACBOW0198	*Triticum aestivum* cv. Chinese Spring	*Aegilops longissima* 4/7S^l^ addition	tall floral ^1^
TACBOW0204	*Triticum aestivum* cv. Chinese Spring	*Aegilops searsii* 4S^s^ addition	tall floral ^1^
TACBOW0206	*Triticum aestivum* cv. Chinese Spring	*Aegilops searsii* 6S^s^ addition	dwarfed
TACBOW0209	*Triticum aestivum* cv. Chinese Spring	*Aegilops longissima* ?S^l^ addition	tall floral
TACBOW0214	*Triticum aestivum* cv. Chinese Spring	*Agropyron elongatum* 3ES addition	dwarfed
TACBOW0215	*Triticum aestivum* cv. Chinese Spring	*Agropyron elongatum* 3EL addition	tall floral ^1^
TACBOW0217	*Triticum aestivum* cv. Chinese Spring	*Agropyron elongatum* 6ES addition	dwarfed
TACBOW0219	*Triticum aestivum* cv. Chinese Spring	*Agropyron elongatum* 6EL addition	dwarfed
TACBOW0220	*Triticum aestivum* cv. Chinese Spring	*Elymus trachycaulus* T1H^t^S·2H^t^S addition	dwarfed
TACBOW0221	*Triticum aestivum* cv. Chinese Spring	*Elymus trachycaulus* T1H^t^S·3S^t^L monosomic addition	tall floral
TACBOW0222	*Triticum aestivum* cv. Chinese Spring	*Elymus trachycaulus* T1H^t^S·6H^t^L addition	dwarfed
TACBOW0224	*Triticum aestivum* cv. Chinese Spring	*Elymus trachycaulus* T1S^t^L·7S^t^L addition	tall floral ^1^
TACBOW0226	*Triticum aestivum* cv. Chinese Spring	*Elymus trachycaulus* T5H^t^L·5H^t^L addition	tall floral
TACBOW0230	*Triticum aestivum* cv. Chinese Spring	*Elymus trachycaulus* T1H^t^S·5H^t^L addition	dwarfed
TACBOW0232	*Triticum aestivum* cv. Chinese Spring	*Aegilops peregrina* T3U^v^#1–? addition	tall floral ^1^
TACBOW0235	*Triticum aestivum* cv. Chinese Spring	*Secale cereale* 2R addition	dwarfed
TACBOW0236	*Triticum aestivum* cv. Chinese Spring	*Secale cereale* 3R addition	tall floral
TACBOW0239	*Triticum aestivum* cv. Chinese Spring	*Secale cereale* 6R addition	dwarfed
TACBOW0244	*Triticum aestivum* cv. Chinese Spring	*Aegilops longissima* 4S^l^ addition	tall floral
TACBOW0245	*Triticum aestivum* cv. Chinese Spring	*Aegilops longissima* 5S^l^ addition	dwarfed
TACBOW0246	*Triticum aestivum* cv. Chinese Spring	*Aegilops longissima* 6S^l^ addition	dwarfed
TACBOW0250	*Triticum aestivum* cv. Chinese Spring	*Elymus trachycaulus* 1H^t^L addition	tall floral ^1^
TACBOW0252	*Triticum aestivum* cv. Chinese Spring	*Elymus trachycaulus* 1S^t^ addition	dwarfed
TACBOW0254	*Triticum aestivum* cv. Chinese Spring	*Elymus trachycaulus* 6H^t^ addition	dwarfed
TACBOW0256	*Triticum aestivum* cv. Chinese Spring	*Elymus trachycaulus* 7H^t^S addition	tall floral ^1^
TACBOW0259	*Triticum aestivum* cv. Chinese Spring	*Elymus trachycaulus* 5H^t^S addition	dwarfed
TACBOW0260	*Triticum aestivum* cv. Chinese Spring	*Elymus trachycaulus* 5H^t^ addition	tall floral
TACBOW0261	*Triticum aestivum* cv. Chinese Spring	*Elymus trachycaulus* 5S^t^ addition	tall floral
TACBOW0262	*Triticum aestivum* cv. Chinese Spring	*Elymus trachycaulus* 5S^t^S addition	dwarfed
TACBOW0265	*Triticum aestivum* cv. Chinese Spring	*Elymus ciliaris* 1Y^c^ addition	dwarfed
TACBOW0267	*Triticum aestivum* cv. Chinese Spring	*Aegilops longissima* ?S^l^ addition	tall floral ^1^
TACBOW0272	*Triticum aestivum* cv. Chinese Spring	*Aegilops peregrina* 4S^v^ addition	tall floral
TACBOW0273	*Triticum aestivum* cv. Chinese Spring	*Aegilops peregrina* 5S^v^ addition	tall floral ^1^
TACBOW0275	*Triticum aestivum* cv. Chinese Spring	*Aegilops peregrina* 1U^v^ addition	tall floral
TACBOW0278	*Triticum aestivum* cv. Chinese Spring	*Aegilops peregrina* 3U^v^ substitution	tall floral ^1^
TACBOW0279	*Triticum aestivum* cv. Chinese Spring	*Aegilops peregrina* 4U^v^ addition	tall floral ^1^
TACBOW0280	*Triticum aestivum* cv. Chinese Spring	*Aegilops peregrina* 5U^v^ addition	dwarfed
TACBOW0282	*Triticum aestivum* cv. Chinese Spring	*Aegilops peregrina* 7U^v^ addition	tall floral ^1^
TACBOW0283	*Triticum aestivum* cv. Chinese Spring	*Aegilops geniculata* 1M^g^ addition	dwarfed
TACBOW0288	*Triticum aestivum* cv. Chinese Spring	*Aegilops geniculata* 6M^g^ addition	tall floral ^1^
TACBOW0290	*Triticum aestivum* cv. Chinese Spring	*Aegilops geniculata* 1U^g^ addition	tall floral ^1^
TACBOW0291	*Triticum aestivum* cv. Chinese Spring	*Aegilops geniculata* 2U^g^ addition	dwarfed
TACBOW0293	*Triticum aestivum* cv. Chinese Spring	*Aegilops geniculata* 5U^g^ addition	dwarfed
TACBOW0295	*Triticum aestivum* cv. Chinese Spring	*Aegilops geniculata* 3U^g^ addition	tall floral ^1^
TACBOW0299	*Triticum aestivum* cv. Chinese Spring	*Elymus ciliaris* ?Y^c^ addition	dwarfed

^1^ delayed maturation.

## Data Availability

The TACBOW lines are available from the National BioResouce Project (NBRP)-Komugi: https://shigen.nig.ac.jp/wheat/komugi/. URL accessed on 27 May 2024.

## Supplementary Materials

The following supporting information can be downloaded at: https://www.mdpi.com/article/10.3390/jof10060384/s1, Table S1. Wheat lines used in study; Table S2.
