# Supplementary material for: Alien Chromatin from Hordeeae Grasses Enhances the Compatibility of Epichloë Endophyte Symbiosis with the Hexaploid Wheat Triticum aestivum
Source: J Fungi (Basel). 2024 May 27;10(6):384. doi: 10.3390/jof10060384 (PMC11204924; doi:10.3390/jof10060384)
Supplement: Supplementary file 1 [file jof-10-00384-s001.zip › Simpson JoF Supplementary Table S1 .pdf]

Supplementary Table S1. Wheat lines used in study.

**Wheat line**	**Alien chromosome**
Chinese Spring	
Monad	
TACBOW0001	*Leymus racemosus* A addition
TACBOW0003	*Leymus racemosus* E addition
TACBOW0004	*Leymus racemosus* F addition
TACBOW0005	*Leymus racemosus* H addition
TACBOW0006	*Leymus racemosus* I addition
TACBOW0007	*Leymus racemosus* J addition
TACBOW0008	*Leymus racemosus* K addition
TACBOW0009	*Leymus racemosus* L addition
TACBOW0010	*Leymus racemosus* N addition
TACBOW0011	*Leymus racemosus* H substitution
TACBOW0012	*Leymus racemosus* 2Lr#1 addition
TACBOW0013	*Leymus racemosus* 5Lr#1 addition
TACBOW0014	*Leymus racemosus* 7Lr#1 addition
TACBOW0015	*Leymus racemosus* 7Lr#1 addition
TACBOW0016	*Leymus racemosus* ?Lr#1 addition
TACBOW0017	*Leymus racemosus* 2Lr#1 addition
TACBOW0018	*Secale cereale* 1R addition
TACBOW0020	*Secale cereale* 3R addition
TACBOW0024	*Secale cereale* 7R addition
TACBOW0025	*Secale cereale* 1R addition
TACBOW0026	*Secale cereale* 1R addition
TACBOW0027	*Secale cereale* 2R addition
TACBOW0028	*Secale cereale* 3R addition
TACBOW0029	*Secale cereale* 5R addition
TACBOW0032	*Aegilops intermedium* 7Ai addition
TACBOW0034	*Aegilops intermedium* 1Ai addition
TACBOW0035	*Aegilops intermedium* 4Ai addition
TACBOW0036	*Aegilops intermedium* 5Ai addition
TACBOW0038	*Aegilops elongatum* 1E addition
TACBOW0039	*Aegilops elongatum* 2E addition
TACBOW0040	*Aegilops elongatum* 3E addition
TACBOW0041	*Aegilops elongatum* 4E addition
TACBOW0042	*Aegilops elongatum* 6E addition
TACBOW0043	*Aegilops elongatum* 5E addition
TACBOW0044	*Aegilops elongatum* 7E addition
TACBOW0045	*Aegilops umbellulata* 1U addition
TACBOW0046	*Aegilops umbellulata* 2U addition
TACBOW0047	*Aegilops umbellulata* 4U addition
TACBOW0048	*Aegilops umbellulata* 5U addition

TACBOW0049	*Aegilops umbellulata* 6U addition
TACBOW0051	*Hordeum chilense* 1Hch substitution
TACBOW0052	*Hordeum chilense* 2HchS addition
TACBOW0053	*Hordeum chilense* 4Hch addition
TACBOW0054	*Hordeum chilense* 5Hch addition
TACBOW0055	*Hordeum chilense* 6Hch addition
TACBOW0056	*Hordeum chilense* 7Hch addition
TACBOW0057	*Haynaldia villosa* 1V addition
TACBOW0059	*Haynaldia villosa* 3V addition
TACBOW0060	*Haynaldia villosa* 4V addition
TACBOW0061	*Haynaldia villosa* 5V addition
TACBOW0062	*Haynaldia villosa* 6V addition
TACBOW0064	*Triticum aestivum-Secale cereale* amphidiploid
TACBOW0065	*Triticum aestivum-Secale cereale* amphidiploid
TACBOW0066	*Triticum turgidum-Secale cereale* amphidiploid
TACBOW0067	*Triticum turgidum-Secale cereale* amphidiploid
TACBOW0068	*Triticum turgidum-Haynaldia villosa* amphidiploid
TACBOW0069	*Triticum aestivum-Aegilops intermedium* amphidiploid
TACBOW0070	*Triticum aestivum-Aegilops intermedium* amphidiploid
TACBOW0123	*Leymus mollis* A addition
TACBOW0124	*Leymus mollis* G addition
TACBOW0125	*Leymus mollis* H addition
TACBOW0126	*Psathyrostachys huashanica* A addition
TACBOW0127	*Psathyrostachys huashanica* B addition
TACBOW0128	*Psathyrostachys huashanica* C addition
TACBOW0133	*Aegilops intermedium* B addition
TACBOW0136	*Aegilops intermedium* E addition
TACBOW0137	*Aegilops intermedium* F addition
TACBOW0138	*Aegilops intermedium* G addition
TACBOW0189	*Aegilops caudata* B addition
TACBOW0190	*Aegilops caudata* C addition
TACBOW0191	*Aegilops caudata* D addition
TACBOW0193	*Aegilops caudata* E addition
TACBOW0195	*Aegilops longissima* 1S^l^ addition
TACBOW0196	*Aegilops longissima* 2S^l^ addition
TACBOW0197	*Aegilops longissima* 3S^l^ addition
TACBOW0198	*Aegilops longissima* 4/7S^l^ addition
TACBOW0201	*Aegilops searsii* 1S^s^ addition
TACBOW0202	*Aegilops searsii* 2S^s^ addition
TACBOW0204	*Aegilops searsii* 4S^s^ addition
TACBOW0205	*Aegilops searsii* 5S^s^ addition
TACBOW0206	*Aegilops searsii* 6S^s^ addition
TACBOW0208	*Aegilops longissima* ?S^l^ addition
TACBOW0209	*Aegilops longissima* ?S^l^ addition

TACBOW0213	*Aegilops elongatum* 1ES addition
TACBOW0214	*Aegilops elongatum* 3ES addition
TACBOW0215	*Aegilops elongatum* 3EL addition
TACBOW0217	*Aegilops elongatum* 6ES addition
TACBOW0219	*Aegilops elongatum* 6EL addition
TACBOW0220	*Elymus trachycaulus* T1H^t^S·2H^t^S addition
TACBOW0221	*Elymus trachycaulus* T1H^t^S·3S^t^L monosomic addition
TACBOW0222	*Elymus trachycaulus* T1H^t^S·6H^t^L addition
TACBOW0224	*Elymus trachycaulus* T1S^t^L·7S^t^L addition
TACBOW0225	*Elymus trachycaulus* T2H^t^S·5H^t^L addition
TACBOW0226	*Elymus trachycaulus* T5H^t^L·5H^t^L addition
TACBOW0227	*Elymus trachycaulus* T7AL·1AS-1S^t^S substitution
TACBOW0228	*Elymus trachycaulus* T7AL·1AS-1S^t^S (7A) & T5DL·7AS (5D) translocation
TACBOW0229	*Elymus trachycaulus* T1H^t^S·1H^t^S addition
TACBOW0230	*Elymus trachycaulus* T1H^t^S·5H^t^L addition
TACBOW0231	*Elymus trachycaulus* T1H^t^S·7S^t^L addition
TACBOW0232	*Aegilops peregrina* T3U^v^#1–? addition
TACBOW0233	*Elymus trachycaulus* T1H^t^S·1BL translocation
TACBOW0235	*Secale cereale* 2R addition
TACBOW0236	*Secale cereale* 3R addition
TACBOW0237	*Secale cereale* 4R addition
TACBOW0239	*Secale cereale* 6R addition
TACBOW0240	*Secale cereale* 7R addition
TACBOW0242	*Aegilops longissima* 2S^l^ addition
TACBOW0244	*Aegilops longissima* 4S^l^ addition
TACBOW0245	*Aegilops longissima* 5S^l^ addition
TACBOW0246	*Aegilops longissima* 6S^l^ addition
TACBOW0247	*Aegilops longissima* 2S^l^ addition
TACBOW0249	*Elymus trachycaulus* 1H^t^S addition
TACBOW0250	*Elymus trachycaulus* 1H^t^L addition
TACBOW0252	*Elymus trachycaulus* 1S^t^ addition
TACBOW0253	*Elymus trachycaulus* 5H^t^ addition
TACBOW0254	*Elymus trachycaulus* 6H^t^ addition
TACBOW0256	*Elymus trachycaulus* 7H^t^S addition
TACBOW0257	*Elymus trachycaulus* 7S^t^L addition
TACBOW0258	*Elymus trachycaulus* 1S^t^L addition
TACBOW0259	*Elymus trachycaulus* 5H^t^S addition
TACBOW0260	*Elymus trachycaulus* 13H^t^ addition
TACBOW0261	*Elymus trachycaulus* 5S^t^ addition
TACBOW0262	*Elymus trachycaulus* 5S^t^S addition
TACBOW0264	*Elymus ciliaris* 1S^c^ addition
TACBOW0265	*Elymus ciliaris* 1^Y^ addition
TACBOW0266	*Elymus ciliaris* 1Y^c^S addition
TACBOW0267	*Aegilops longissima* ?S^l^ addition

TACBOW0268	*Aegilops longissima* ?S^l^ addition
TACBOW0270	*Aegilops peregrina* 2S^v^ addition
TACBOW0271	*Aegilops peregrina* 3S^v^ addition
TACBOW0272	*Aegilops peregrina* 4S^v^ addition
TACBOW0273	*Aegilops peregrina* 5S^v^ addition
TACBOW0274	*Aegilops peregrina* 7S^v^ addition
TACBOW0275	*Aegilops peregrina* 1U^v^ addition
TACBOW0276	*Aegilops peregrina* 2U^v^ addition
TACBOW0277	*Aegilops peregrina* 2Uv^ addition & 2BL deletion
TACBOW0278	*Aegilops peregrina* 3U^v^ addition
TACBOW0279	*Aegilops peregrina* 4U^v^ addition
TACBOW0280	*Aegilops peregrina* 5U^v^ addition
TACBOW0281	*Aegilops peregrina* 6U^v^ addition
TACBOW0282	*Aegilops peregrina* 7U^v^ addition
TACBOW0283	*Aegilops geniculata* 1M^g^ addition
TACBOW0284	*Aegilops geniculata* 2M^g^ addition
TACBOW0286	*Aegilops geniculata* 4M^g^ addition
TACBOW0287	*Aegilops geniculata* 5M^g^ addition
TACBOW0288	*Aegilops geniculata* 6M^g^ addition
TACBOW0290	*Aegilops geniculata* 1U^g^ addition
TACBOW0291	*Aegilops geniculata* 2U^g^ addition
TACBOW0292	*Aegilops geniculata* 4U^g^ addition
TACBOW0293	*Aegilops geniculata* 5U^g^ addition
TACBOW0295	*Aegilops geniculata* 3U^g^ addition
TACBOW0296	*Elymus ciliaris* 2S^c^ addition
TACBOW0297	*Elymus ciliaris* 3S^c^ addition
TACBOW0298	*Elymus ciliaris* 1S^c^ & 5Y^c^ double addition
TACBOW0299	*Elymus ciliaris* ?Y^c^ addition
