# Supplementary material for: Alien Chromatin from Hordeeae Grasses Enhances the Compatibility of Epichloë Endophyte Symbiosis with the Hexaploid Wheat Triticum aestivum
Source: J Fungi (Basel). 2024 May 27;10(6):384. doi: 10.3390/jof10060384 (PMC11204924; doi:10.3390/jof10060384)
Supplement: Supplementary file 1 [file jof-10-00384-s001.zip › Simpson JoF Supplementary Table S2.pdf]

**Line**	**Addition**
TACBOW 011	*Leymus racemosus* H substitution
TACBOW 053	*Hordeum chilense* 4Hch addition
TACBOW 197	*Aegilops longissima* 3S l addition
TACBOW 232	*Aegilops peregrina* T3U addition
TACBOW 288	*Aegilops geniculata* 6M g addition
